# Effects of *Rhizophora mangle* on Experimental Colitis Induced by TNBS in Rats

**DOI:** 10.1155/2012/753971

**Published:** 2012-09-27

**Authors:** Felipe Meira de Faria, Anderson Luiz-Ferreira, Eduardo Augusto Rabelo Socca, Ana Cristina Alves de Almeida, Ricardo José Dunder, Luis Paulo Manzo, Marcelo Aparecido da Silva, Wagner Vilegas, Ariane Leite Rozza, Cláudia Helena Pellizzon, Lourdes Campaner dos Santos, Alba Regina Monteiro Souza Brito

**Affiliations:** ^1^Departamento de Farmacologia, Faculdade de Ciências Médicas, Universidade Estadual de Campinas (UNICAMP), 13083-970 Campinas, SP, Brazil; ^2^Departamento de Biologia Estrutural e Funcional, Instituto de Biologia, UNICAMP, 13083-970 Campinas, SP, Brazil; ^3^Departamento de Química Orgânica, Instituto de Química, UNESP, 14800-900 Araraquara, SP, Brazil; ^4^Departamento de Morfologia, Instituto de Biociências, UNESP, 18618-970 Botucatu, SP, Brazil

## Abstract

Male Unib-WH rats were pretreated for two weeks with butanolic (BuOH) and ethyl acetate (EtOAc) fractions. Colitis was induced by rectal administration of TNBS, the treatment continued, and animals were sacrificed on day 7 after the TNBS administration. Phytochemical studies were performed in order to provide the characterization of the tannins present in the bark of *R. mangle*. Results showed that EtOAc fraction increased the levels of IL-10 (∗∗*P* < 0.01) and diminished the levels of TNF-**α** (∗∗∗*P* < 0.001) and IL-6 (∗∗*P* < 0.01). BuOH fraction reduced the MPO activity (∗∗*P* < 0.01) and levels of TBARS (∗∗∗*P* < 0.001); it also increased COX-1 expression, diminished the levels of TNF-**α** (∗∗∗*P* < 0.001), and increased the levels of IL-12 (∗∗∗*P* < 0.001). Besides, both treatments augmented the levels of GSH (∗*P* < 0.05), the activity of GSH-Px (∗∗*P* < 0.01 for BuOH fraction and ∗∗∗*P* < 0.001 for EtOAc fraction), and CAT (∗∗*P* < 0.01). In conclusion, both treatments ameliorated the injury induced by TNBS through different mechanisms, probably by their chemical composition which directed its activity into an antioxidant or anti-inflammatory response, leading to an immune modulation.

## 1. Introduction

Inflammatory bowel diseases (IBDs) such as ulcerative colitis (UC) and Crohn's disease (CD) have complex etiologies but are simply characterized by chronic active intestinal inflammation that waxes and wanes. The main features of these diseases are a robust inflammatory response of unknown origin associated with mucosal injury and increased epithelial permeability, invasion of commensal bacteria into the subepithelial space or lamina propria, and massive recruitment of neutrophils [1].

The UC primarily affects the mucosal lining of the colon and rectum, whereas CD usually affects the whole intestinal wall and potentially may extend to any part of the gastrointestinal tract [2]. Intestinal homeostasis requires a controlled innate immune response to the microbiota, which is recognized by toll-like receptors (TLRs) and nucleotide-binding oligomerization domain- (NOD-) like receptors on the epithelial and immune cells. This recognition process contributes to tolerance, but when the process is dysregulated, inflammation ensues. In CD, abnormalities of innate immunity are linked to variants of receptor genes, the products of which normally mediate microbial recognition [3].

Various proinflammatory mediators such as cyclooxygenases (COX-1 and COX-2), tumor necrosis factor-alpha (TNF-*α*), interleukin-6 (IL-6), and interleukin-12 (IL-12), the presence of highly activated inflammatory cells such as neutrophils, dendritic cells, macrophages, and excessive production of reactive oxygen species (ROS) have been implicated in the pathogenesis of this disease [4]. 

Many polyphenolic compounds have been studied for their intestinal anti-inflammatory activity, whereas the main effect of these compounds on inflammatory conditions is due to the inhibition of proinflammatory markers [5, 6]. In this context, medicinal plants which accumulate polyphenolic compounds have an important role in the investigation of novel therapeutics agents.

*Rhizophora mangle* is a mangrove plant which is a rich source of phenolic compounds, especially condensed and hydrolysable tannins; its bark is known as a traditional medicine in Brazil and Caribbean countries [7]. In recent years, various authors have described some activities such as antioxidant [8], anti-inflammatory [9], wound healing [10] and antiulcer [7, 11]. The present work aimed at evaluating the effects of *Rhizophora mangle* extracts on the experimental TNBS-induced colitis in rats. 

## 2. Material and Methods

### 2.1. Animals

Unib-WH male rats (180–250 g) obtained from the breeding of the Universidade Estadual de Campinas (CEMIB/UNICAMP) were used. Animals were fed with a certified Nuvilab CR-diet, with free access to tap water, and were housed on a 12 h light/dark cycle at 60 ± 1% humidity and a temperature of 21 ± 2°C.

### 2.2. Plant Material and Preparation of the Extracts

The barks of *Rhizophora mangle* L. were collected in “Estuário de Santos,” Santos, SP, Brazil. Professor Msc. Paulo Salles Penteado Sampaio authenticated the botanical identity of the plants, and a voucher specimen (HUSC-P.S.P. Sampaio et al., 800) was deposited to the (Herbário da Universidade Santa Cecília HUSC). The preparation of the extracts of the bark of *Rhizophora mangle* as well as their phytochemical analysis was performed according to de-Faria et al. [11].

### 2.3. FIA-ESI-IT-MS

The *fingerprints* were obtained in a mass spectrometer LCQ Fleet (Therma Analitica), equipped with a direct injection dispositive via continuous flow injection analysis (FIA). ESI *fingerprints* in the negative ion mode of *R. mangle* extract and fractions were acquired and accumulated over 60 s and spectra were scanned in a range between *m/z* 50 and 1200, and multiple fragments (MS^2^, MS^3^) were performed in an ion-trap interface (IT). Capillary and spray voltages were set at −4 V and −5 kV, respectively, with a desolvation temperature of 280°C, carrier gas (N_2_) flow rate 60 (arbitrary units). Firstly, a *full-scan fingerprint* was obtained, after that, MS^*n*^ experiments were made from the first scan of the preselected precursor ions with collision energy between 25% and 30% of total energy of the equipment.

The Xcalibur software version 1.0 (Therma Scientific) was used during the acquisition and spectrometric data processing.

### 2.4. Experimental Colitis

Male Unib-WH rats were randomly assigned to four groups (*n* = 8); two of them (noncolitic and control groups) received no treatment, and the others received, orally, daily for 3 weeks, 0.5 mg/Kg of BuOH fraction, 1.5 mg/Kg of EtOAc fraction (suspended in saline 10 mL/kg). Both noncolitic and control groups (colitic nontreated) were given daily 10 mL/kg of saline. Two weeks after the treatment started, the rats were fasted overnight and those from the control and treated groups were rendered colitis by the method originally described by Morris et al. [12]. Briefly, they were anaesthetized with halothane and given 10 mg of TNBS dissolved in 0.25 mL of 50% ethanol (v/v) through a Teflon cannula inserted 8 cm through the anus. Rats from the noncolitic group were administered intracolonically with 0.25 mL of phosphate-buffered saline instead of TNBS. Behavior, body weight, and stool consistency were recorded daily throughout the experiment. All rats were killed with an overdose of halothane 1 week after induction of colitis, and the colon was removed aseptically and placed on an ice cold plate and longitudinally opened. 

### 2.5. Assessment of Colonic Damage

For each animal, the distal 10 cm portion of the colon was removed, slightly cleaned in physiological saline for faecal residues removal, and weighed. Macroscopic inflammation scores were assigned based on clinical features of the colon (score 0–10: 0 (no damage), 1 (focal hyperaemia), 2 (ulceration without hyperaemia or bowel wall thickening), 3 (ulceration with inflammation at 1 site), 4 (≥2 sites of ulceration and inflammation), 5 (major sites of inflammation >1 cm along the organ), 6–10 (major sites of inflammation >2 cm along the organ)). Stool consistency (score 0-1) was evaluated according to the criteria of Bailón et al. [13] and Vicario et al. [14]. Pieces of inflamed colon were collected and subsequently divided into longitudinal segments and frozen in liquid nitrogen for further measurement of biochemical parameters.

#### 2.5.1. Antioxidant Assay

**(1) Levels of Glutathione (GSH)**

GSH levels of colonic tissue of animals were determined by Ellman's reaction using 5′5′-dithio-bis-2-nitrobenzoic acid (DTNB) as described by Anderson [15]. The intensity of the yellow colour was read at 412 nm.

**(2) Glutathione Peroxidase Activity (GSH-Px)**

GSH-Px activity was quantified by following the decrease in absorbance at 365 nm induced by 0.25 mM H_2_O_2_ in the presence of reduced glutathione (10 mM), NADPH, (4 mM), and 1 U enzymatic activity of GSH-Px [16].

**(3) Catalase (CAT)**

Catalase activity was performed according to the method described by Aebi [17]. The samples were homogenized (1 : 200) in phosphate buffer (50 mM, ph 7.0) and triton x-100 (9 : 1). Then, samples were centrifuged (7000 g, 4°C, 15 minutes); for 200 *μ*L of the homogenate were added 100 *μ*L of H_2_O_2_ (30 mM). Absorbance was measured at 240 nm for 2 minutes. The catalase activity was expressed as U/mg of protein, and U of enzyme activity is defined as the amount of enzyme required to degrade 1 *μ*mol of H_2_O_2_/s/mg of protein.

**(4) Myeloperoxidase Activity (MPO)**

MPO activity in the colonic mucosa was measured by the method proposed by Krawisz et al. [18] to evaluate neutrophil accumulation. The tissue was minced with hexadecyltrimethylammonium bromide (HTAB) buffer on ice and homogenized for three times (3 s) on ice; the homogenizer was rinsed twice with 1 mL of HTAB, the homogenate and washes were sonicated for 10 s, freeze-thawed three times, then, aliquots of homogenate were mixed with a reaction buffer of 50 mM phosphate buffer, pH 6.8, containing 0.005% H_2_O_2_ and 1.25 mg/mL o-dianisidine dihydrochloride and then measured at 460 nm.

**(5) Estimation of Lipid Peroxidation (TBARS)**

The homogenate of the colon was diluted in 0.15 M KCl (ratio 1 : 10). Then, to 0.5 mL of this homogenate, 0.2 mL of dodecyl sulfate (8.1%), 1.5 mL of acetic acid (20%, adjusted with NaOH solution to pH 3.5), 1.5 mL thiobarbituric acid (0.8% w/v), and 0.3 mL of distilled water were added. All samples were left in water bath set at 95°C for 1 hour. After this period, the samples were cooled and added to 1 mL of distilled water and 5 mL of the mixture n-butanol : pyridine (15 : 1, v/v), shaken in vortex for 1 min and centrifuged at 1400 G for 10 minutes. The absorbance of organic layer was determined at 532 nm. TEPP (1,1,3,3-tetraethoxypropane) diluted in ethanol was used as standard. The results were expressed as picomoles of substances that react with thiobarbituric acid (TBARS) per mg of protein (nmol TBARS/mg protein) [19].

#### 2.5.2. Determination of the Cytokine Levels

Tissue homogenate was centrifuged, and the supernatant was stored at −70°C. Cytokine detection was performed by enzyme-linked immunosorbent assay (ELISA) for TNF-*α*, IL-6 (DY 406), IL-10 (DY 522), and IL-12 (p70) (DY 419) with the Duoset kit (R&D Systems, Minneapolis, MN, USA).

#### 2.5.3. Western Blotting Analysis

Frozen colon samples were homogenized in 1 mL of cold buffer containing phosphate buffer-PB 0.1 M, pH 7.4, and protease inhibitor cocktail-1% (Sigma-Aldrich P-8340). Homogenates were centrifuged (12,000 ×g, 15 min, 4°C), and the supernatants were collected and stored at −80°C. Protein concentration of the homogenate was determined following Bradford's colorimetric method [20]. Then, samples were treated with Laemmili buffer (PB 0.5 M, pH 6.8; glycerol, sodium dodecyl sulfate (SDS) 10%, bromophenol 0.1%, *β*-mercaptoethanol) in a 1 : 1 proportion. Equal amounts of protein from samples (100 *μ*g) were separated on 10% acrylamide gel by sodium dodecyl sulfate polyacrylamide gel electrophoresis. In the next step, proteins were electrophoretically transferred onto a nitrocellulose membrane and incubated with specific primary antibodies: COX-1(160109, Cayman Chemical, USA) and COX-2 (160126, Cayman Chemical, USA) at 1 : 500 dilution. Each membrane was washed three times for 10 min and incubated with HRP-Goat Anti-Rabbit (Invitrogen 656120) (for COX-1 and COX-2, all diluted at 1 : 5000). To prove equal loading, the blots were analyzed with standard protein dye ponceau [21]. Immunodetection was performed using enhanced chemiluminescence light-detecting kit (SuperSignal West Femto Chemiluminescent Substrate, Pierce, IL, USA). Densitometric data were performed with G-BOX, Syngene following normalization to the control (ponceau) by GeneSys software. 

## 3. Histology

The slides were observed after haematoxylin and eosin (HE) staining. Histological analyses were made using a Leica microscope associated with Leica Q-Win Software 3.1 (Leica-England), from the Image Analysis Laboratory of the Department of Morphology, UNESP Botucatu.

## 4. Statistical Analysis

Results were expressed as mean ± standard error of means (SEM). The statistical significance of each test group in relation to the control was calculated using ANOVA followed by Dunnet or Tukey's *t*-test.

## 5. Results

The FIA-ESI-ITMS/MS technique with sample direct injection has been used as a quick, efficient, and sensitive tool. It is capable of generating specific information about the chemical composition of matrix complex, which presents substances with a broad range of molecular weight, as well as medium and high polarity substances, such as phenolic acids, proanthocyanidins, catechins, and flavonoids, commonly found in polar extracts of plants [22].

Regarding these characteristics, FIA-ESI-IT-MS/MS assays were made to obtain and compare the *fingerprints* of the acetone : water 7 : 3 extract and active fractions, Butanolic (BuOH fraction) and Ethyl Acetate (EtOAc fraction), in negative mode, which allows us to establish and confirm the similarity (or not) of the chemical composition of these extracts and fractions.

The investigation of the *full scan* mass spectra reveals the presence of various substances, which are represented by the precursor ions of the deprotonated molecules ([M–H]^−^) (Table 1) for the extract and fractions obtained from the barks of *Rhizophora mangle.*

The ion at *m/z* 289 was assigned to the deprotonated molecule of (epi)catechin whose fragmentation led to the base peak at *m/z* 137, which represents C-ring cleavage through a retro-Diels-Alder (RDA) mechanism [23, 24]. A minor product ion at *m/z *271 was due to the loss of water with double-bond formation (Table 1). The chirality of the C3 on the flavan-3-ols cannot be differentiated by mass spectrometry. Designations of the epimer configuration are generally based on the relative abundance of the “epi” configuration as polyphenolic components of tea infusions and extracts [25, 26]. In our case, we designated the compounds as [epi] catechins. 

**Figure 1:**
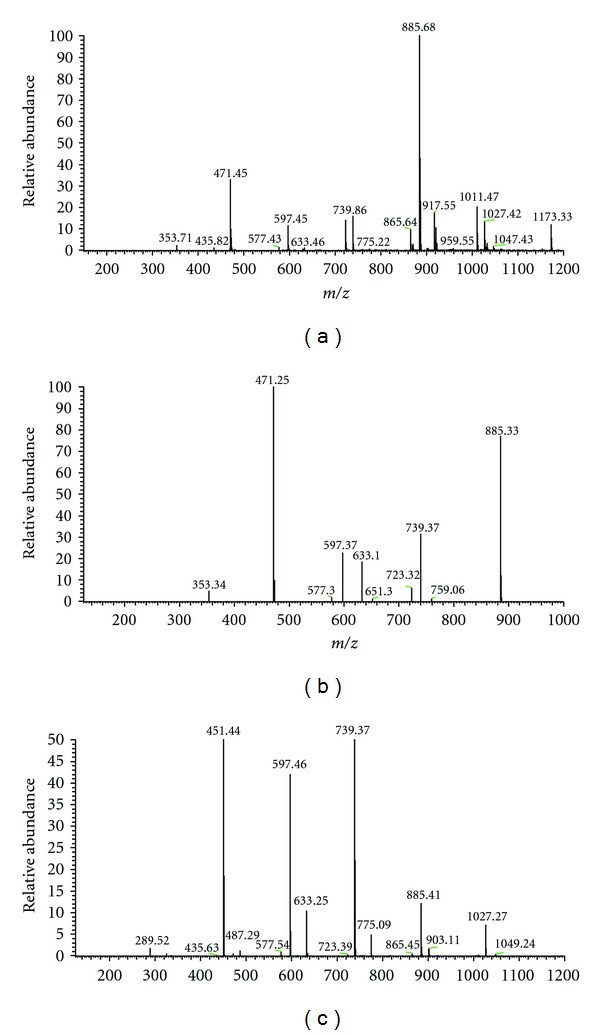
Direct flow injection ESI-IT-MS *fingerprint* spectra in *full scan*, obtained in negative ion mode. (a) Normal mass scan in the range of *m/z* 50–1200 of the acetone : water (7 : 3, v/v) extract, (b) normal mass scan in the range of *m/z* 50–1200 of the BuOH fraction, and (c) normal mass scan in the range of *m/z* 50–1200 of the EtOAc fraction. For spectrometric conditions, see Section 2.

**Figure 2:**
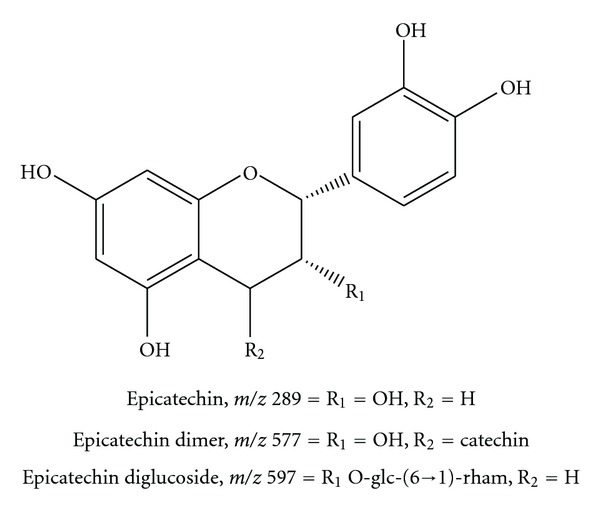
Epicatechins identified in the barks from *R. mangle* by FIA-ESI-IT-MS/MS, negative mode.

In a first class of secondary metabolites, the precursor ion of (epi)catechin formed by interflavonoidic ligation between catechin units of *m/z* 577 (dimer), [M–H]^−^, was found in the *full scan* of the extract (Figures 1(a), 1(b), and 1(c), Table 1, Figure 2). Another catechin derivative, comprising two hexoses, was identified on *m/z* 597. The spectrum MS^2^ provided a loss of 146 Da probably due to the loss of a terminal rhamnose, affording the ion *m/z* 451 (Table 1, Figure 2).

**Figure 3:**
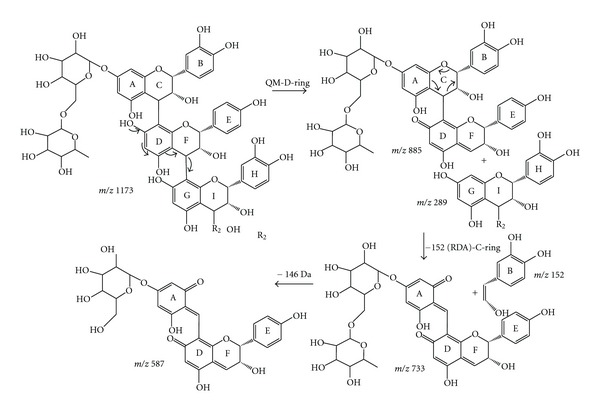
Main fragmentation pathways proposed of a trimer proanthocyanidin identified in the extract acetone : water (7 : 3, v/v) and BuOH fraction and EtOAc fraction from the barks of *R. mangle*. The mechanisms principal: RDA (retro-Diels-alder) and QM (quinone methide).

 Fragmentation MS/MS of precursor ion of *m/z* 1173 (Figure 1(a)) produced base peak of *m/z* 885; this ion was generated by the elimination of one unit of (epi)catechin [M–288–H]^−^ of the (epi)catechin unit of this trimer (Table 1, Figure 3).

In order to verify the results described above, MS^3^ experiments for the main observed peaks were performed. The MS^3^ of ion on *m/z* 773 refers to a loss of a 152 Da [M–288–152–H]^−^ formed due to RDA fragmentation (Figure 3). Another consistent proposal was used to explain the presence of the ion on *m/z* 587, generated by the mechanism of quinone-methide (QM) fragmentation, occurring on D-ring of the superior unit with loss of residuum 1,3,5-trihydroxybenzene glucoside sequence for the elimination of the resultant fragment of retro-Diels-alder (RDA) on the C-ring of the central unit [M–288–152–H]^−^ (Table 1, Figure 3).

On the mechanism of fragmentation involving the QM cleavage, the localization of diglucoside on the superior unit of the deprotonated molecule was postulated based on a cleavage of the interflavonoidic ligation between the C-ring of the superior unit and the D-ring of the central unit (Figure 3). The evidence that the glucose is in the superior unit of the trimer is given by the ion *m/z* 885. This ion is the result of an elimination of the lower unit of catechin (G-I-rings), with consequent formation of the fragment in which superior unit is a diglucoside catechin (A, B and C-rings) and the lower unit containing a QM system (D-, E-, and F-rings). Such fragment is impossible to be released if the glucose is in the lower unit of the trimer.

 Proanthocyanidins are exclusively constituted by equivalent units of (epi)catechins that present one interflavonoidic ligation, which are called procyanidins type B. Thus, the precursor ions of *m/z* 577, *m/z* 885, and *m/z* 1173 are indicatives of the procyanidins type-B [27]. However, as in MS techniques, any differentiation was realized, as well as any information about the position and stereochemistry of the interflavonoidic ligation.

 The oral administration of BuOH fraction and EtOAc fraction for 21 days attenuates the macroscopic colon injury caused by the TNBS. Both treatments were capable of diminishing the extension of the lesions, although only BuOH fraction provided significant changes in adhesion, extension of lesions, and score. 

 BuOH fraction and EtOAc fraction maintained the levels of GSH at physiological patterns, as well as the activity of GSH-Px and Catalase; moreover, the GSH-Px activity was found increased in both treatments.

 Only BuOH fraction exerts effects on the MPO activity and on lipid peroxidation in colonic tissue, reducing the MPO activity and TBARS levels.

 The treatments were capable of modulating the levels of cytokines in inflamed colon, EtOAc fraction diminished the levels of proinflammatory cytokines TNF-*α*, IL6, and IL-12, and it also augmented IL-10 levels, an anti-inflammatory cytokine; BuOH fraction decreased the levels of TNF-*α* and IL12.

**Figure 4:**
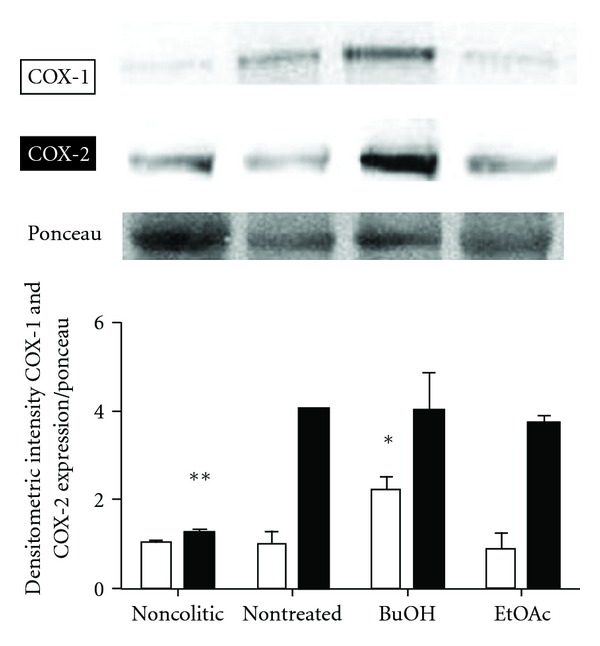
Effects of oral administration during 3 weeks of BuOH fraction and EtOAc fraction on COX-1 and COX-2 expression in colonic tissue of rats submitted to TNBS-induced colitis. Densitometry was made following normalization with ponceau. Results are presented as mean ± SEM ANOVA followed by Dunnett's *t*-test, *P < 0.05 and **P < 0.01 significantly different from nontreated.

The treatments (BuOH fraction and EtOAc fraction) were not capable of decreasing the expression of COX-2 (Figure 4). COX-1 expression was found at physiological levels in all groups, except BuOH fraction treatment which showed an increased expression (Figure 4).

**Figure 5:**
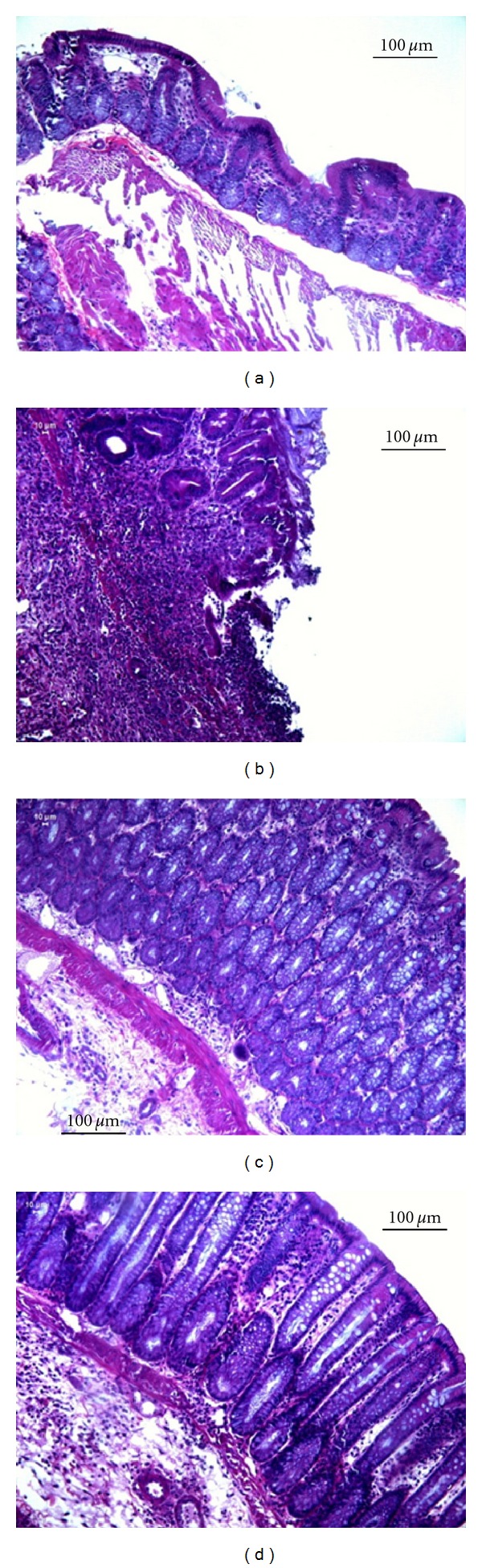
Histological slices of the colon from rats submitted to TNBS-induced colitis assay. HE staining shows comparison of the tissue architecture between treatments: (a) noncolitic, (b) colitic nontreated, (c) BuOH fraction, and (d) EtOAc fraction.

 The representative histological evaluation shows normal colonic mucosa (Figure 5(a)) compared with colitic animals: nontreated (Figure 5(b)), BuOH fraction (Figure 5(c)), and EtOAc fraction (Figure 5(d)). Despite a nonquantitative analysis, it is remarkable that BuOH fraction presents the mucosa without marked reduction of inflammatory infiltrate. 

## 6. Discussion

 This study focused on the beneficial effects of the BuOH fraction and EtOAc fraction of the bark of *Rhizophora mangle *in TNBS-induced chronic colitis in rats. *R. mangle* has been studied in our laboratory in the recent years, and we have demonstrated its antiulcer [11] and antioxidant [8] effects in previous works. In the present work, we continued *R. mangle* study based on its benefits to treat gastrointestinal diseases; thus, we selected both fractions and the respective active doses (0.5 mg/Kg—BuOH fraction and 1.5 mg/Kg—EtOAc fraction) found in the previous studies cited. 

Here, we showed the protective effects of both fractions in an experimental model of colitis, where the treatment with fractions of *R. mangle* results in the amelioration of the colonic mucosa injury (Table 2), the improvement of antioxidant contents (GSH) and enzymes (GSH-Px, catalase) as well as the modulation of inflammatory markers (MPO, TBARS, COX-1, and COX-2) and cytokines (TNF-*α*, IL-6, IL-12, and IL-12).

The *fingerprint* of the *R. mangle* acetone : water (7 : 3, v/v) extract and BuOH fraction and EtOAc fraction obtained by FIA-ESI-ITMS/MS corroborate with the first proposal of the presence of tannins from the polymerization of catechins.

The suggested fragmentations for ions of some identified molecules reveal the presence of proanthocyanidin and its possible derivatives O-glucoside. A more comprehensive discussion for another precursor ions was recently reported by our group [27] where the FIA-ESI-IT-MS analysis helped to solve the chemical composition of the phytopreparation complex. With this *fingerprint* of *R. mangle,* it is also possible to verify the similarity between the extract and the active fractions, probably by the presence of procyanidins.

The TNBS model of colitis induces a transmural lesion with pathological characteristics resembling human Crohn's disease; in this context and in order to evaluate the significance of the therapeutic use of the procyanidins from the bark of *Rhizophora mangle,* the protocol of TNBS-induced colitis in rats was used. 

The production of proinflammatory cytokines, such as interleukin-6, interleukin-12, and tumor necrosis factor (TNF-*α*), is universally increased in patients with inflammatory bowel disease. Abnormalities of adaptive immunity that differentiate ulcerative colitis from Crohn's disease are defined by mucosal CD4+ T cells, which were initially divided in two lineages: Th1 and Th2 T cells. Crohn's disease is a Th1-like condition [3].

 The characteristic inflammatory response begins with an infiltration of neutrophils and macrophages, which then release chemokines and cytokines. Neutrophil infiltration into the inflamed mucosa is one of the most prominent histological features observed in IBD. Activated neutrophils produce reactive oxygen and nitrogen species within intestinal mucosa inducing an oxidative stress, which plays a significant role in the pathogenesis of IBD [28]. Regarding this knowledge, our results show that treatment with BuOH fraction, but not EtOAc fraction, decreased MPO activity (Table 4). This could be related to the lower injury found in this group, although this finding is linked to the evaluation of other ROS mediators, as shown in the following.

 Inflammatory mediators, including reactive oxygen species (ROS) and cytokines, contribute to the inflammatory cascade, modulating the immune response in IBD; TNF-*α* can activate oxidative stress-responsive genes which amplify and prolong inflammation [29]. Several studies have shown that ROS play an important role in the pathogenesis of IBD; excessive production of ROS in mucosal cells induces an immediate inflammatory immune response which directly or indirectly enhances the damage to intestinal epithelial cells, subsequently, harming the mucosal integrity, leading to severe injury in the gut [30, 31]. 

Oxidative stress and its consequent lipid peroxidation could aggravate free radical chain reactions, disrupt the integrity of the intestinal mucosal barrier, and activate inflammatory mediators. It has been shown that colonic CAT and GSH-Px activity and GSH levels are decreased while lipid peroxidation (TBARS levels) is increased in experimental and clinical studies [32, 33]. 

 CAT, GSH-Px, and GSH, as primary defenses, could reduce the oxidative stress and the activation of inflammatory mediators; *R. mangle* acting as free radical scavenger could counteract the function of ROS and directly scavenge hydroxyl radicals and peroxyl radical species [31]. In our study, compared with the noncolitic group, the activities of CAT and GSH-Px with GSH levels in colon tissues dwindled remarkably in the colitic nontreated group. Therapy with BuOH fraction or EtOAc fraction for 21 days resulted in a marked increase in CAT and GSH-Px activities (Table 3) in colon tissues; however, only BuOH fraction treatment was capable of reducing TBARS levels (Table 4). In addition, these treatments resulted in colonic GSH levels (Table 4) increase. Our results suggested that both BuOH fraction and EtOAc fraction inhibited oxidative stress induced by TNBS. The beneficial effects of these treatments are due, partly, to the antioxidant capacity of the compounds presented in its composition. 

Recently, we published a study on the antioxidant effects of BuOH fraction in experimental models of gastric ulcer [8]; the study shows that BuOH fraction can protect the gastric mucosa through the enhancement of antioxidant mediators and enzymes, such as GSH, GSH-Px, GSH-Rd, SOD as well as diminishing MPO activity and lipid peroxidation. Thus, the previous results are in accordance with the present data regarding the different tissues and pathogenesis between the respective models.

 The expression of COX-2, but not COX-1, is stimulated after the induction of colitis by TNBS [34], although Hyun et al. [35] showed that in PAR_2_-deficient mice, COX-1 and COX-2 have their expression augmented after the induction of colitis by TNBS, and this was associated with a reduction in inflammation. Thus, during the development of colitis, both isoforms of COX could together exert an anti-inflammatory effect. For Reuter et al. [36], COX-1 and COX-2 have an active role in modulating the inflammatory process in various experimental models of colitis, since the administration of selective inhibitors of COX-1 or COX-2 in models of TNBS and DSS-induced colitis increased the severity of colitis and harmed the healing process. In this context, our results (Figure 4) suggest that BuOH fraction exerts its anti-inflammatory through a similar mechanism since the expression of both COX-1 and COX-2 was increased in this group, while EtOAc fraction presented similar levels of expression of these enzymes found in colitic nontreated group. 

 Cytokines are essential mediators of the interactions between activated immune cells and nonimmune cells, including epithelial and mesenchymal cells. The clinical efficacy of TNF-*α* clearly indicates that cytokines are one of the therapeutic targets of chronic inflammatory disorders such as IBD [37]. 

TNF-*α* is a major mediator of inflammation in the gut. It is synthesized by several cells including intestinal epithelial cells but predominantly by cells of the monocytic line and T lymphocytes. TNF-*α* induces the expression of various genes; it has multiple biological effects such as increasing leukocyte recruitment, modulation of nitric oxide production, induction of proinflammatory cytokine secretion, and the proliferation and differentiation of immune cells [3].

Our results showed that both treatments BuOH fraction and EtOAc fraction provided downregulation of TNF-*α* levels (Table 5), suggesting that these compounds could act on the activation of this cytokine. Interestingly BuOH fraction and EtOAc fraction also decreased the levels of IL-12 (Table 5), which are linked to the suppression of TNF-*α*; this fact may possibly explain this mechanism by which both treatments ameliorate the inflammation scenario. Anti-IL-12 or interferon-*γ*-specific antibodies antagonize the development of spontaneous IBD in IL-10-deficient mice, but only neutralization of IL-12 ameliorated the disease [4]. IL-12 blockade inhibits the generation of Th1 response and abrogates the production of the downstream cytokines (T-cell derived TNF-*α*, IFN-*γ*, and IL-12 itself). 

Overproduction of IL-12, a macrophage-derived cytokine, shifts the immune response in a Th-1direction; this response is characterized by increased production of TNF-*α* and IL-6 resulting in a self-sustaining cycle of activation [38]. IL-6 is a pleiotropic cytokine that plays a crucial role in inflammation, immune regulation, hematopoiesis, and oncogenesis; there is growing evidence that an IL-6 signaling pathway plays a crucial part in the uncontrolled intestinal inflammatory process of IBD [39]. The evaluation of this proinflammatory cytokine revealed that EtOAc fraction decreased its level; however, BuOH fraction showed no effects on its levels.

These proinflammatory cytokines act on local cell populations to promote intracellular killing (superoxides, peroxides), enhance recruitment of other inflammatory cells, enhance secretion of chemoattractant cytokines (chemokines), and promote local tissue destruction [40]. Mucosal dendritic cells (DCs), in particular, have long been thought to promote tolerance in the gut. Expanding dendritic cells, including those present in the intestinal tissues, promote the ability to induce oral tolerance. Moreover, mucosal DCs residing in the lamina propria or Peyer's patches of the intestine appear to preferentially produce IL-10, rather than IL-12 [41]. 

IL-10 is secreted by a wide variety of cells and has pleiotropic effects on T cells, B cells, myeloid cells, and other cell types. IL-10 has suppressive anti-inflammatory activity on T cells, macrophages, and dendritic cells in humans, as well as in animal models of inflammatory diseases. Despite that IL-10 effectively treats colitis in mouse models and suppresses inflammatory cytokine production *in vivo *in intestinal cells from IBD patients [3], in diseases with a relative or absolute IL-10 deficiency, a persistent immune activation still exists [42]. Regarding the evaluation of IL-10 (Table 5), the treatments showed different responses again, while EtOAc fraction increased its level, and BuOH fraction showed no effect on IL-10 level. 

This finding suggests that the main effect of the compounds of EtOAc fraction is likely to act on the activation of anti-inflammatory IL-10 and on the downregulation of IL-12, thus resulting in diminished levels of TNF-*α* and IL-6. On the other hand, the compounds present in BuOH fraction possibly exhibited their anti-inflammatory activity in this model by the regulation of cyclooxygenases as well as the activity and levels of ROS mediators, especially the lipid peroxidation (TBARS) and MPO which are also linked to neutrophil infiltration, thereby promoting healing changes during the inflammation caused by TNBS.

## 7. Conclusions

The compounds present in BuOH fraction and EtOAc fraction are predominantly proanthocyanidins and procyanidins; these compounds have shown interesting activities as mentioned previously. In this work, we provided, for the first time, the evaluation of these compounds from *R. mangle *barks on intestinal inflammation as a continuous study on the benefits of this medicinal plant regarding the gastrointestinal tract. Here, we showed a promising evaluation on anti-inflammatory and immune response of these compounds, and this study must be used for further studies to help explaining how these compounds lead to these subtle mechanisms such as activation of IL-10, COX-1, and other related cells and molecules.

On the structural elucidation of the compounds present in the bark of *R. mangle*, further studies must be continued by our research group, where the extract and active fractions will be fractionated by classic chromatographic methods for isolation of substances and identification by spectrometric methods, in trying to obtain a chemical marker for the extract.

## Figures and Tables

**Table 1:** ESI-MS and ESI-MS^*n*^ product ions of proanthocyanidins and procyanidins occurring in the bark of *R. mangle*.

[M–H]^−^	Product ions
289	137 [M–152–H]^−^, 271 [M–18–H]^−^
577	289 [M–288–H]^−^, 137 [M–152–H]^−^, 271 [M–18–H]^ −^
597	451 [M–146–H]^−^
1173	885 [M–288–H]^−^, 733 [M–288–152–H]^−^, 587 [M–288–152–146–H]^−^

**Table 2:** Effects of BuOH fraction and EtOAc fraction on parameters observed in colon of rats submitted to colitis induced by TNBS.

Group	*n*	Score	Lesion (cm)	Weigh/length (mg/cm)	Adhesion	Diarrhea
Noncolitic	6	0***	0***	91.6 ± 2.5***	0***	0***
Colitic nontreated	10	9.2 ± 0.6	5.5 ± 0.8	225.6 ± 56.7	1.5 ± 0.5	1.0 ± 0.0
BuOH fraction 0.5 mg/Kg	10	8.1 ± 0.8*	4.3 ± 0.8*	217.8 ± 18.0	0.4 ± 0.6**	0.7 ± 0.4
EtOAc fraction 1.5 mg/Kg	9	8.3 ± 1.0	4.4 ± 1.3*	227.3 ± 40.2	1.0 ± 0.7	0.7 ± 0.4

Results are presented as mean ± SEM ANOVA followed by Dunnett's *t*-test, *P < 0.05, **P < 0.01, ***P < 0.001 significantly different from colitic nontreated.

**Table 3:** Effect of BuOH fraction and EtOAc fraction on colonic GSH level, GSH-Px, and catalase activities in TNBS-induced colitis in rats.

Group	Colon GSH levels (*μ*mol/mg of protein)	Colon GSH-Px activity (nmol/min/mg of protein)	Colon CAT activity (U/mg of protein)
Noncolitic	8.320 ± 1.720**	11.070 ± 0.638*	17.510 ± 3.198**
Colitic nontreated	1.028 ± 1.026	4.472 ± 0.409	1.459 ± 1.082
BuOH fraction 0.5 mg/Kg	6.548 ± 0.935*	11.620 ± 0.869**	15.910 ± 2.409**
EtOAc fraction 1.5 mg/Kg	6.706 ± 1.969*	14.850 ± 1.573***	15.660 ± 3.380**

Results are presented as mean ± SEM ANOVA followed by Dunnett's *t*-test, *P < 0.05, **P < 0.01, ***P < 0.001 significantly different from colitic nontreated.

**Table 4:** Effect of BuOH fraction and EtOAc fraction on colonic MPO activity and TBARS levels in TNBS-induced colitis in rats.

Group	Colon MPO activity (U/mg of protein)	Colon TBARS levels (*μ*mol TBARS/mg of protein)
Noncolitic	0.642 ± 0.061**	4.294 ± 0.707**
Colitic nontreated	2.891 ± 0.740	5.737 ± 0.139
BuOH fraction 0.5 mg/Kg	0.896 ± 0.102**	4.171 ± 0.106***
EtOAc fraction 1.5 mg/Kg	2.264 ± 0.429	7.218 ± 0.776

Results are presented as mean ± SEM ANOVA followed by Dunnett's *t*-test, *P < 0.05, **P < 0.01, ***P < 0.001 significantly different from colitic nontreated.

**Table 5:** Effect of BuOH fraction and EtOAc fraction on colonic TNF-*α*, IL-6, IL12, and IL-10 levels in TNBS-induced colitis in rats.

Group	Colon TNF-*α* levels (pg/mg of protein)	Colon IL-6 levels (pg/mg of protein)	Colon IL-12 levels (pg/mg of protein)	Colon IL-10 levels (pg/mg of protein)
Noncolitic	357.3 ± 20.7***	209.1 ± 24.3***	12.7 ± 1.9***	295.0 ± 37.7***
Colitic nontreated	1381.0 ± 227.5	807.3 ± 45.5	28.5 ± 2.4	120.4 ± 4.3
BuOH fraction 0.5 mg/Kg	469.5 ± 21.5***	719.1 ± 17.4	16.72 ± 1.0***	152.5 ± 11.6
EtOAc fraction 1.5 mg/Kg	567.9 ± 21.8***	615.4 ± 54.4**	19.09 ± 1.7**	252.1 ± 19.3**

Results are presented as mean ± SEM ANOVA followed by Dunnett's *t*-test, *P < 0.05, **P < 0.01, ***P < 0.001 significantly different from colitic nontreated.
